# Structural validity of the Brazilian version of the quality care questionnaire-palliative care for use in individuals with diabetes mellitus eligible for palliative care

**DOI:** 10.1186/s13098-024-01548-w

**Published:** 2024-12-19

**Authors:** Abraão Albino Mendes-Júnior, Aldair Darlan Santos-de-Araújo, Leonel Richard de Oliveira Silva Santos, Lorena Lúcia Costa Ladeira, Meire Coelho Ferreira, Louise Aline Romão Godim, Mariana Campos Maia, Marinete Rodrigues de Farias Diniz, Almir Vieira Dibai-Filho, Daniela Bassi-Dibai

**Affiliations:** 1 Postgraduate Program in Management and Health Care, Ceuma University, Rua Josué Montello, 1, Jardim Renascença, São Luís, MA 65075-120 Brazil; 2 Cardiopulmonary Physiotherapy Laboratory, Federal University of São Carlos, São Carlos, SP Brazil; 3 Nursing Department, Ceuma University, São Luís, MA Brazil; 4 Postgraduate Program in Dentistry, Ceuma University, São Luís, MA Brazil; 5 Postgraduate Program in Environment, Ceuma University, São Luís, MA Brazil; 6 Physical Therapy Department, Ceuma University, São Luís, MA Brazil; 7 Postgraduate Program in Physical Education, Federal University of Maranhão, São Luís, MA Brazil

**Keywords:** Palliative care, Diabetes mellitus, Validation study, Quality of health care

## Abstract

**Background:**

The Brazilian version of the Quality Care Questionnaire-Palliative Care (QCQ-PC) is an instrument designed to assess the quality of care provided in palliative care from the user’s perspective, featuring easy comprehension and applicability. It has demonstrated validity for use in individuals with cancer, but there is a need for validation in other populations due to the scarcity of instruments with this purpose.

**Objective:**

To structurally validate the Brazilian version of the QCQ-PC for use in individuals with diabetes mellitus (DM) eligible for palliative care.

**Methods:**

This is a structural validation study of a questionnaire according to the Consensus-based Standards for the Selection of Health Measurement Instruments (COSMIN). The study was conducted with 100 individuals with DM. Data collection occurred in differents care services in São Luís (northeast Brazil) by means of the application of the QCQ-PC and a form with sociodemographic, clinical, and daily habit data. Descriptive data analysis was performed using absolute values, relative frequencies, and measures of central tendency and dispersion. Structural validity was assessed by means of exploratory factor analysis (EFA).

**Results:**

Of the 100 participants included in the study, 66% were female, 54% were single, with a median age of 64 years, 44% were overweight (44%), 77% were on polypharmacy, and 70% were physically inactive. We found that one domain is the most appropriate for use of the QCQ-PC in individuals with DM eligible for palliative care, according to the parallel analysis implemented in the EFA. This domain was named “quality of care”. The fit indices for this one-dimensional internal structure were adequate: Kaiser–Meyer–Olkin test = 0.71, p value < 0.01 in Bartlett’s test, chi-square/degree of freedom = 1.07, comparative fit index = 0.993, Tucker-Lewis index. = 0.991, root mean square error of approximation = 0.028. The QCQ-PC presented factor loadings ranging from 0.480 to 0.883, maintaining a total of 12 items, which demonstrates the adequate relationship between the quality of care domain and its items.

**Conclusion:**

Therefore, the internal structure with one domain (quality of care) is the most suitable for use in individuals with DM eligible for palliative care by means of the QCQ-PC.

## Introduction

Historically, the concept of palliative care was defined as an approach whose objective was to alleviate suffering in the final stage of life. However, today, this concept has been remodeled so that it can be implemented earlier in those individuals whose health condition poses risks to life, regardless of the disease [1]. It is estimated that, worldwide, 56.8 million individuals need this type of care [1]. Among them are those affected by Non-Communicable Diseases (NCDs), a group of diseases that primarily affects low- and middle-income countries [2, 3]. In Brazil, NCDs accounted for 74% of deaths in 2016, affecting different social strata, being prevalent in the North and Northeast regions, and among poorer populations [4–6]. Diabetes Mellitus (DM) is one of the four main groups of NCDs, estimated to affect around 8.6% of Brazilian population and to have caused 65.000 deaths in 2018 [7–10]. Additionally, DM is associated with premature death, increased hospitalizations, and a greater need for care [11, 12].

People with diabetes have the right to receive information about palliative care and end-of-life care options, which should be integrated into diabetes management and education as function and disease trajectory evolve. However, these topics, especially in the early stages of diabetes, have recently been proactively introduced into educational programs or clinical consultations for these individuals and their families [13, 14]. Life expectancy is shorter in people with DM than in the general population due to complications and comorbidities. In this context, palliative and end-of-life care are essential but neglected aspects of comprehensive and personalized DM treatment. Early initiation of palliative care and proactive end-of-life planning have been recommended, as they improve symptom management, comfort, and quality of life. In addition, they reduce disease burden and unnecessary treatment [14, 15].

The aforementioned scenario contributes to the worsening of the clinical condition and, consequently, the well-being of this population, contrary to the recommendation that care should be offered as early as possible to prevent complications [16, 17]. Furthermore, it complicates the measurement of the quality of care received by populations receiving or eligible for palliative care, given the lack of validated instruments to assess the quality of care provided [18]. This metric is necessary to understand the services offered, allowing for an assessment of the strengths and weaknesses of the services, thus generating feedback for continuous improvement and, consequently, enhancing the quality of care delivered to the population [18]. Additionally, it is known that low-quality or inadequate instruments result in biased data, failing to contribute to knowledge and potentially posing risks depending on their use [18].

Among the available instruments specifically designed to assess the quality of palliative care provided, there is the Quality Care Questionnaire-Palliative Care (QCQ-PC), an instrument created in South Korea for use with individuals diagnosed with cancer receiving palliative care [19]. It consists of 32 items with four response options [19]. In its original version, it is composed of four domains: (1) appropriate communication with healthcare professionals, (2) discussion about the value of life and care goals, (3) support and guidance for comprehensive care needs, and (4) accessibility and sustainability of care. The instrument demonstrates consistency and validity for convergent applicability [19]. In light of this, a cross-cultural adaptation was carried out for the Brazilian population, resulting in two domains: (1) communication with health professionals; (2) care and assistance provided by health professionals, and 12 items with four response options, showing reliability, adequacy, instrumental validity, as well as easy applicability and good comprehension for use in individuals receiving palliative care with an oncological diagnosis [20].

However, the instrument was validated only for use with the oncological population [20]. Thus, there is a need for validation for other populations in palliative care or even those eligible to receive such care. In this context, this study aimed to structurally validate the Brazilian version of the QCQ-PC for use in individuals with DM eligible for palliative care.

## Methodology

### Study design

This is a structural validation study of a questionnaire in individuals with DM, following the criteria of the COnsensus-based Standards for the Selection of Health Measurement INstruments (COSMIN) [21]. The study was conducted in three primary care services, one secondary care service and one tertiary care service in São Luís (Maranhão, Northeast Brazil) from August to December 2023. The research was approved by the Ethics Committee of the Ceuma University (report number: 6.159.628). All individuals invited to participate were informed about the study’s objectives, methodological procedures, risks, benefits, and ethical aspects related to the research. They confirmed their participation by voluntarily signing the informed consent.

### Participants

The sample size was determined using the rule of 7 times the number of items in the questionnaire. Considering that the QCQ-PC has 12 items, the measured sample size is 84. However, the rule defines a minimum participation of 100 individuals [21]. The study included individuals with DM who were eligible for palliative care and were being followed up by different care services (primary, secondary and tertiary care) in São Luís. For palliative care eligibility, individuals were selected who self-reported at least three indicators of health deterioration and correspond to factors associated with increased morbidity and mortality in diabetic patients: decline, presence of macrovascular and/or microvascular complications (diabetic retinopathy, diabetic peripheral neuropathy with clinical diagnosis, lower limb amputation), functional dependence in decline, weight loss > 10% in the last 6 months, polypharmacy (using 4 or more medications), occurrence of hospitalizations associated with DM and complications, glycemic instability (hyperglycemia and hypoglycemia), depression and presence of other comorbidities[15].

In addition to the mentioned characteristics, participants had to be at least 18 years old and have been in follow-up for at least 3 months. Individuals who did not fully respond to the instrument were excluded. Patients were invited to participate while waiting for their appointment or during visits conducted by two trained researchers (A.A.M.J and L.R.O.S.) and community health workers. The characterization of the study participants was carried out through sociodemographic, clinical, and daily habits data.

### The Brazilian Version of the Quality Care Questionnaire-Palliative Care (QCQ-PC)

The Brazilian version of the QCQ-PC consists of 12 items and 2 domains: “communication with healthcare professionals” and “care and assistance provided by healthcare professionals”. Each item has four response options, respectively: strongly agree (score 4), agree (score 3), disagree (score 2), and strongly disagree (score 1) [20]. The score calculation is performed separately for each domain, corresponding to the sum of all answered items, followed by subtraction by 6, and finally divided by 0.18, with the result ranging from 0 to 100. A higher score reflects greater satisfaction with the quality of care provided by the healthcare team, while a lower score indicates greater dissatisfaction [20].

### Statistical analysis

Descriptive analysis was performed using mean and standard deviation or median and interquartile range for numerical variables, and percentage for categorical variables, with the purpose of characterizing the study participants. Data normality was analyzed via Kolmogorov–Smirnov test.

Due to the lack of evidence regarding the internal structure of the Brazilian version of the QCQ-PC for a non-oncological population, the internal structure was analyzed by means of exploratory factor analysis (EFA) [22, 23]. Initially, a polychoric matrix and a robust diagonally weighted least squares (RDWLS) extraction method were used, since the response options for each QCQ-PC item are ordinal values [22, 23].

The retention of factors was determined by means of parallel analysis implementation in the EFA, with random permutation of the observed data, and the rotation used was robust promin [24, 25]. Data processing was performed using the FACTOR software (Universitat Rovira i Virgili, Tarragona, Spain) [26]. The model’s adequacy was evaluated using the Kaiser–Meyer–Olkin (KMO) criterion and Bartlett’s test of sphericity. A KMO value above 0.70 and a significant p value (< 0.05) in Bartlett’s test were considered adequate indices [27, 28]. In addition to these indices, we also used the following fit indices to define the model as adequate: values greater than 0.90 were considered adequate for comparative fit index (CFI) and Tucker-Lewis index (TLI); value less than 0.08 was considered adequate for root mean square error of approximation (RMSEA); value below 3.00 was considered adequate for the interpretation of chi-square/degree of freedom (DF) [29]. Factor loadings equal to or greater than 0.40 were considered adequate for the domain.

## Results

**Fig. 1:**
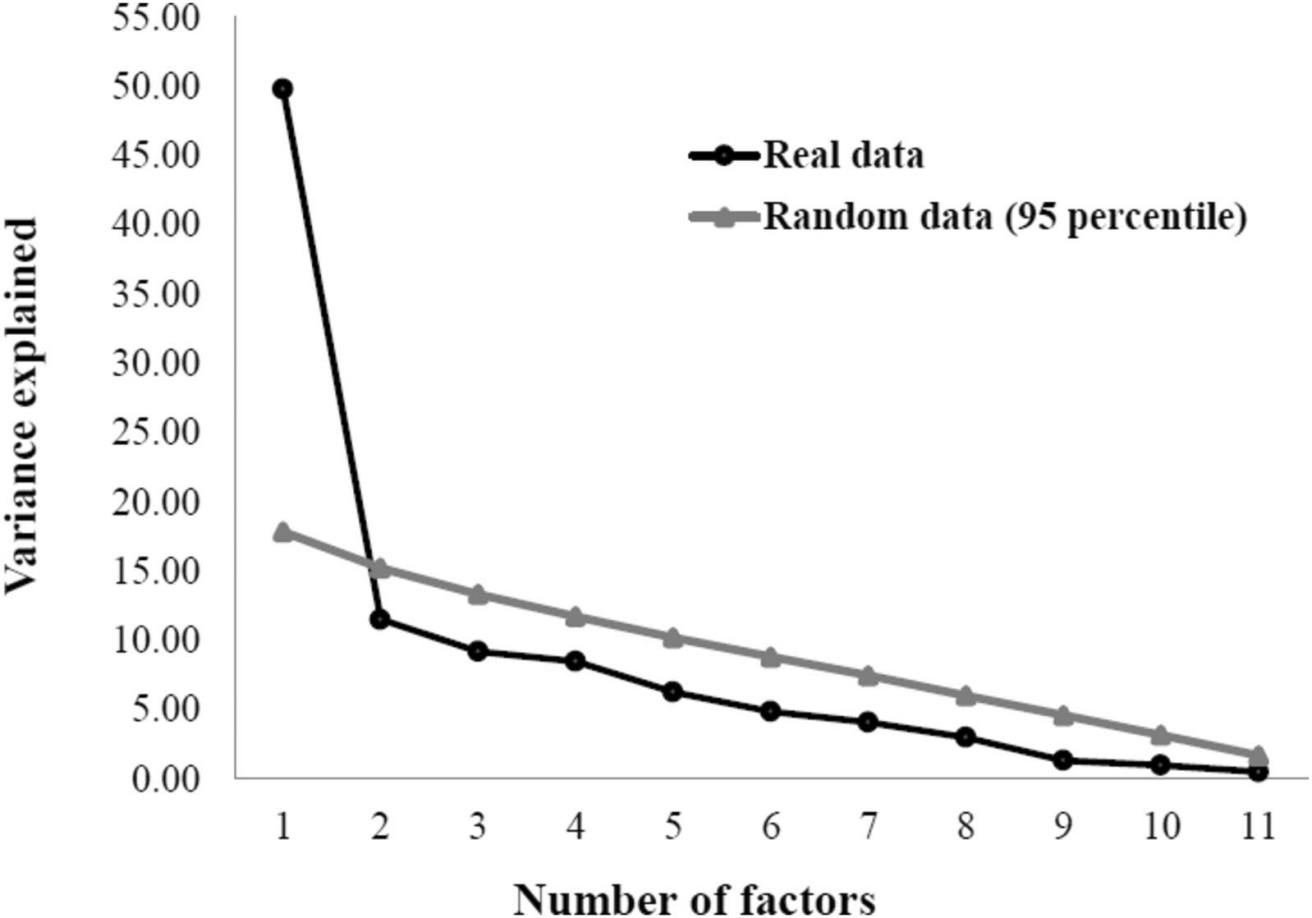
Parallel analysis of the Quality Care Questionnaire-Palliative Care (QCQ-PC) in a sample of individuals with diabetes mellitus

A total of 100 individuals with a diagnosis of diabetes mellitus (DM) participated in the study. As shown in Table 1, 66% were female, 54% were single, with a median age of 64 years, 44% were overweight, 77% were on polypharmacy, and 70% were physically inactive. Regarding comorbidities, 81% of participants had systemic arterial hypertension, 70% had neuropathy and 49% had retinopathy. As shown in Fig. 1, only one domain was identified in the parallel analysis, referred to here as “quality of care”. This structure one-dimensional presented values within acceptable ranges for all fit considered in this study: KMO = 0.71, p value < 0.01 in Bartlett’s test, chi-square/DF = 1.07, CFI = 0.993, TLI = 0.991, RMSEA (90% confidence interval) = 0.028 (0.000 to 0.036).

**Table 1:** Sample Characteristics (n = 100)

Characteristic	Values
Age (years)^a^	64.00 (14.75)
Time since diagnosis (years)^a^	10.50 (13.00)
Weight (kg)^a^	66.00 (14.13)
Height (m)^b^	1.54 (0.18)
Body mass index^c^	
Underweight (< 18.5 kg/m^2^)	1%
Normal weight (18.5 to 25 kg/m^2^)	29%
Overweight (25 to 30 kg/m^2^)	44%
Obese (> 30 kg/m^2^)	26%
Sex^c^	
Female	66%
Male	34%
Marital status^c^	
Single	54%
Married	41%
Divorced	3%
Widower	2%
Comorbidities^c^	
Retinopathy (yes)	49%
Neuropathy (yes)	70%
Amputation (yes)	15%
Nephropathy (yes)	8%
High blood pressure (yes)	81%
Dyslipidemia (yes)	46%
Heart disease (yes)	12%
Others comorbidities (yes)	44%
Hospitalizations due to DM and complications^c^	
Yes	20%
No	80%
Polypharmacy^c^	
Yes	77%
No	23%
Smoking^c^	
Yes	4%
No	96%
Practice of physical activity^c^	
Yes	30%
No	70%
QCQ-PC (score, 0–100)^a^	61.11 (13.89)

*DM* Diabetes Mellitus, *QCQ-PC* Quality Care Questionnaire-Palliative Care

^a^Values presented as median (interquartile range)

^b^Values presented as mean (standard deviation)

^c^Values presented as percentage

Table 2 presents the factor loadings of the relationship between domain and items. A satisfactory factor loading above 0.40 (range: 0.480 to 0.883) was identified for all items of the QCQ-PC. Thus, to calculate the total QCQ-PC score, the following calculation must be performed: sum of all answered items, followed by subtraction by 12, and finally divided by 0.36, with the result ranging from 0 to 100 (higher scores indicate better quality of care).

**Table 2:** Factor loadings of the Quality Care Questionnaire-Palliative Care (QCQ-PC) with their respective confidence intervals

Item	Factor loadings	CI 95%
1. I am satisfied with the way of communication of health staff	0.709	0.532 to 0.828
2. I have heard and understood an accurate description of the progress of my disease	0.769	0.594 to 0.898
3. The health staff explained terms that I was curious about	0.768	0.588 to 0.892
4. I have heard and understood an accurate description of my care plan	0.661	0.437 to 0.819
5. I was able to discourse with health staff about the value of my life	0.624	0.419 to 0.784
6. My family and I received an education that is helpful to care	0.883	0.800 to 0.964
7. My care plans included the things I was able to try myself	0.548	0.333 to 0.710
8. I was able to modify my care plan when my demand for treatment changed	0.750	0.596 to 0.872
9. The health staff provide support to me and my family to solve spiritual concerns	0.508	0.226 to 0.715
10. The health staff provided support to me and my family to overcome social crisis	0.529	0.141 to 0.734
11. The health staff knew what I wanted	0.480	0.196 to 0.672
12. I was able to get care services at the locations I wanted	0.508	0.248 to 0.704

*CI* Confidence interval

## Discussion

The present study showed that: (i) the most appropriate structure of the QCQ-PC for use in patients with DM eligible for palliative care is with a single domain, called “quality of care”; and (ii) the number of items remained the same, i.e., 12 items. In contrast to the version by Barros et al. [20], the only Brazilian validation of the instrument for cancer patients, which has two domains: (i) communication with healthcare professionals; and (ii) care and assistance provided by healthcare professionals. We used the parallel analysis implemented in EFA to identify the domains of the QCQ-PC since it is considered a robust method by previous studies [30, 31] Parallel analysis compares real data to simulated data to identify dimensionality [31]. In addition, we used several fit indices to investigate the adequacy of the unidimensional structure.

Both versions are short forms, with the version in the present study featuring a simpler structure and score calculation, facilitating its use and interpretation, and reducing the chances of filling errors, which are common in longer instruments [20]. The population of the Brazilian version consisted of individuals with cancer receiving palliative care in a hospital setting [20], while the present sample consists of individuals diagnosed with DM receiving primary, secondary and tertiary care.

The original version has four domains: (i) adequate communication with healthcare professionals; (ii) discussion about the value of life and care goals; (iii) support and guidance for comprehensive care needs; and (iv) accessibility and sustainability of care. Moreover, it is emphasized that the instrument, besides being applied to a different population, is situated in a different cultural context [19]. The care received by most of our sample, in primary and secondary care services, resembles palliative care in some aspects, such as continuous and comprehensive follow-up, focus on improving quality of life and preventing complications, timely identification of health issues, and engagement of family members or caregivers.

In primary care policy, one of the principles is the centrality of care on the individual, built with their participation according to their needs, with the aim of achieving well-being, considering the social and family context in which they are inserted [32]. Similarly, in palliative care, the focus is on the interests and autonomy of the individuals and their support network, aiming to provide the highest possible well-being. Thus, efforts are made for timely diagnosis of health issues and avoiding unnecessary interventions [33].

As an instrument designed to assess the quality of palliative care, there is the FAMCARE scale, created to evaluate the quality of care offered to individuals with terminal cancer and their families. The entire sample of the validation process consisted of individuals and families receiving palliative care, thus it can also be used to assess the quality of this care approach [34].

Similarly to the version of the QCQ-PC identified in the present study, the FAMCARE scale is a short instrument containing 20 items with four response options: very satisfied, satisfied, dissatisfied, and very dissatisfied [34]. It is observed that there are no validated instruments in Brazil aimed at evaluating the quality of palliative care, especially from the users’ perspective. There are, however, instruments that assess the quality of care in other healthcare settings, also cross-culturally validated, such as the Patient Satisfaction with Mental Health Services Scale (SATIS-BR) [35].

The SATIS-BR was validated for Portuguese, aiming to assess the user’s perception of mental health services, and is composed of three domains: relationships with the team, the way users are treated by professionals, and satisfaction with the service’s physical conditions, consisting of 15 items [35]. Similar to the QCQ-PC version in the present study, the scale allows for an understanding of aspects of the service, as perceived by users that need improvement. Therefore, the present version of the QCQ-PC provides greater ease in evaluating the quality of services offered to individuals with DM eligible for palliative care, and due to its simplified structure, any entity involved in care can carry out its application.

This study has limitations that should be mentioned, such as not having a defined clinical approach to palliative care. However, we know that the nature of DM is complex, complicating the determination of when a patient can benefit from palliative care or when they are approaching the end of life, making the prognosis challenging. Furthermore, the culture of palliative care is deeply rooted in cancer patients. Therefore, physicians and other health professionals should learn more about the current indications and eligibility criteria for palliative care for chronic and progressive diseases.

## Conclusion

The Brazilian version of the QCQ-PC recommended for use in individuals with DM eligible for palliative care contains only one domain, maintaining the number of 12 items. This version provides a quick and simplified assessment of service quality, which can be easily integrated into service routines. The information collected through the instrument allows for the complementing of evaluations, enabling the identification of the service’s weaknesses and strengths, thus highlighting where efforts should be directed to offer an efficient service.

## Data Availability

The set of data generated and/or analyzed during the present study are available through the corresponding author upon reasonable request.
